# Extraction, Purification, Characterization, Applications of Chitosan, Plant Gum Polysaccharides, and Other Polysaccharides: A Review

**DOI:** 10.1155/sci5/9919005

**Published:** 2025-10-16

**Authors:** Antony V. Samrot, Etel Faradjeva, Amira Abubakar Mohamed, Tan Chuan Sean, Emmanuel Norbert, Xiao Qi Ng, Chua Yeok Mun, Chin Hooi Sze, Akasha Arif, Lee Si Jie, Jane Cypriyana P. J., Saigeetha S., Lavanya Agnes Angalene J., Kalpana Shree S., Hemlata Senthil Kumar, Keerthika V.

**Affiliations:** ^1^Department of Microbiology, Faculty of Medicine, Manipal University College Malaysia, Jalan Padang Jambu, Bukit Baru, Melaka 75150, Malaysia; ^2^School of Bioscience, Faculty of Medicine, Bioscience and Nursing, MAHSA University, Jalan SP2, Bandar Saujana Putra, Jenjarom 42610, Selangor, Malaysia; ^3^Department of Biotechnology, School of Bio and Chemical Engineering, Sathyabama Institute of Science and Technology, Chennai 600119, Tamil Nadu, India; ^4^Department of Biotechnology, School of Biosciences and Technology, Vellore Institute of Technology, Vellore 632014, Tamil Nadu, India; ^5^Department of Biotechnology, Sri Venkateswara College of Engineering, Pennalur-Sriperumbudur Tk, Tamil Nadu 602117, India

**Keywords:** applications, extraction, polysaccharides, purification

## Abstract

Polysaccharides from plant gum, chitosan from animal origin, and other polysaccharides are biologically active, nontoxic, biodegradable, and biocompatible with a wider range of clinical and general applications. The properties of these biological-derived polysaccharides play a major role in their application. This review outlines the different techniques used in the extraction of polysaccharides from plant and animal sources. The importance of the purity of polysaccharides is crucial; thus, techniques such as deproteination and other chromatography techniques are elaborated. Evaluation of properties of polysaccharides using spectroscopy methods, including UV–Vis spectroscopy, Fourier transform infrared (FTIR) spectroscopy, x-ray diffraction (XRD), thermogravimetric analysis (TGA), and microscopic methods, including scanning electron microscopy (SEM) and transmission electron microscopy, is detailed. These biological polysaccharides have been used in various fields including tissue engineering, nanotechnology, and drug delivery. These applications are elaborately covered in detail in this review.

## 1. Introduction

Biopolymers are composed of repeating monomeric units linked by covalent bonds to form long-chain structures and are highly essential for all life forms [1]. These vital building blocks give various structural and functional properties which allow biopolymers to play crucial roles in biological systems and find wide-ranging applications. Based on their constituent monomers, biopolymers are broadly classified into three major categories: polysaccharides, proteins, and nucleic acids. Bioderived natural polysaccharides are found in a variety of natural origins spanning microorganisms to trees [2, 3] and exhibit remarkable properties such as excellent biocompatibility and biodegradability, making them highly attractive for clinical applications [4–6]. Polysaccharides are high-molecular-weight carbohydrates composed of multiple monosaccharide units linked by glycosidic bonds [7], where these glycosidic bond formations are influenced by enzymatic or chemical reaction. These molecules are vital for physiological processes, including energy storage and production in all living organisms [8].

**Figure 1:**
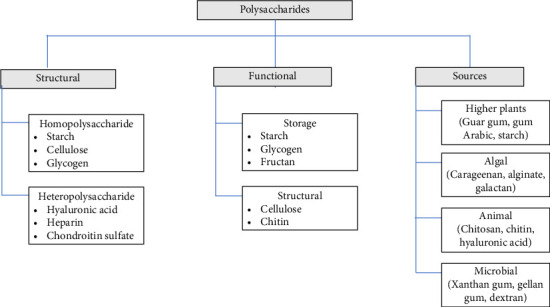
The classification of polysaccharides according to their structure, function, and sources.

Naturally occurring polysaccharides are diverse, categorized by their sources, structures, and functions (Figure 1), and they are classified as homo- or heteropolysaccharides [9]. Examples include plant-derived homopolysaccharides like cellulose and starch [10], and the heteropolysaccharide hyaluronic acid. These polysaccharides are either involved in structure formation (e.g., cellulose in plants, chitin in fungi and arthropods, peptidoglycan in bacterial cell walls) or serve as storage molecules (e.g., starch and glycogen) [11]. Furthermore, they are categorized by their origin, such as higher plants (cellulose) [12], algae (alginate) [13], animals (collagen) [9], and microbes (dextran) [14] (Figure 1). Other notable polysaccharides include mucopolysaccharides (chondroitin sulfate [CS]), pectins, hemicellulose, and peptidoglycan [15].

Their chemical structure and arrangement significantly influence their characteristics and potential [16]. For instance, hemicellulose, a heterogeneous branched polysaccharide, exhibits diverse properties relevant to applications like skin treatments [17], and *Aloe vera* polysaccharides are being exploited for wound healing, antioxidant, and antiaging effects [18]. Bioderived polysaccharides do have therapeutic benefits, including anti-inflammatory, cardiovascular protection, antitumor, antimutation, and antiviral activities [19, 20]. Chitosan-based nanoparticles, for example, are being explored for targeted drug delivery and anti-inflammatory applications in respiratory diseases [21]. Given the global pollution concerns, these biopolymers present ecofriendly alternatives [22], enhancing their clinical and industrial importance.

Plant gum polysaccharides, complex biopolymers from various plant parts [23, 24], are valued for their water solubility, emulsification, biocompatibility, and biodegradability [25], leading to diverse industrial applications. In agriculture, gums from *Moringa oleifera* and *Azadirachta indica* act as plant stimulants and biopesticides [26]. Peach tree gum shows antioxidant and antibacterial properties, making it useful in the food industry [25, 27]. Traditional sources include neem, moringa, flaxseed, tara, locust bean, guar, and tamarind seeds [28]. Plant polysaccharides generally contribute biocompatibility, biodegradability, antioxidant, anti-inflammatory, and antitumor properties [29], with relatively lower manufacturing costs. Advancements have facilitated their use in nanotechnology, bioremediation, biosensors, and biomedicine [30, 31].

Chitosan, the second most abundant biopolymer after cellulose, is derived from the deacetylation of chitin found in marine crustaceans, fungi, and insects [32–34]. Extraction methods involve deproteinization, demineralization, and deacetylation. Utilizing agricultural and forestry waste as substrates for fungal chitosan production can reduce costs [35]. Chitosan's structure, with β-(1-4)-linked *D*-glucosamine units, allows for extensive modifications via its amino and hydroxyl groups [36, 37]. It finds applications in food preservation, drug delivery, wound dressings, scaffolds, and bioremediation [38, 39].

While this review focuses on chitosan and plant gum polysaccharides due to their exceptional biocompatibility, biodegradability, and antimicrobial activity, making them of primary interest in biopolymer-based product development, it is crucial to remember they represent just one class of biopolymers. The extraction, purification, and characterization techniques discussed, along with the broad applications in food, pharmaceuticals, biomedicine, agriculture, and environmental uses, highlight the overall significance of biological polymers as a whole.

## 2. Extraction of Polysaccharides

**Figure 2:**
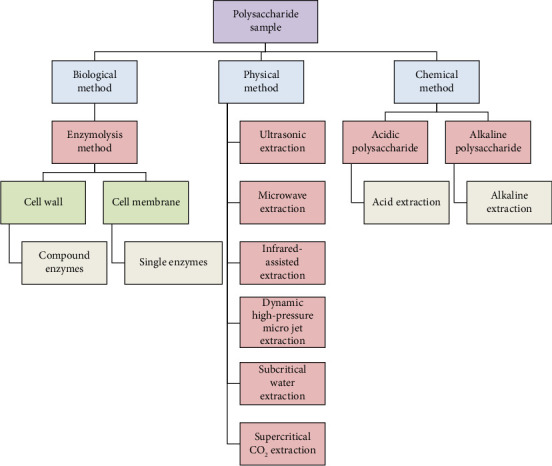
Methods for polysaccharide extraction.

Polysaccharides are generally polar molecules that vary in solubility. While many dissolve in water, others, such as cellulose and chitin, are water-insoluble due to extensive hydrogen bonding. [40]. The main concept of polysaccharides extraction is to disrupt the cell walls by physical or chemical or biological methods or a combination of two or three methods together (Figure 2). The physical methods involve hot extraction, ultrasonic, microwave-assisted extraction, and infrared-assisted extraction. For chemical methods, either alkali or acid is used for the extraction. To minimize polysaccharide degradation, factors like temperature, pH, and extraction time must be carefully optimized. The biological method involves enzymatic hydrolysis [41] where enzymes like cellulase, pectinase, etc., are employed which gently degrade the cell walls, preserving polysaccharide integrity and enhancing yield [42, 43].

### 2.1. Chemical Methods for Polysaccharide Extraction

#### 2.1.1. Alkaline and Acid Extraction

Acidic polysaccharides are characterized mostly to have carboxyl (-COOH) or sulfate (-SO_4_^2-^) groups, which readily get ionized in alkaline solution and form soluble salts. This principle underpins the widespread application of alkaline methods for efficient polysaccharide extraction, particularly from aquatic organisms and plant sources. Notably, alkaline extraction can disrupt glycopeptide linkages present in glycoproteins while concurrently facilitating the solubilization of acidic polysaccharides, thereby contributing to improved extraction efficiency and higher yields [44]. Comparative studies on extraction methods, such as those involving plant gum polysaccharides from *Laminaria japonica*, have highlighted the variability in yielded products across acid, alkaline, and water-based approaches [45]. In one alkaline extraction protocol for *Laminaria japonica*, powdered plant material was treated with a 1% NaOH solution, followed by neutralization with acid, concentration, and precipitation with ethanol. Subsequent steps included deproteination and dialysis [45]. A similar methodology was employed for the acid-mediated extraction of polysaccharides from *Crassostrea rivularis*, where a 1% citric acid solution replaced the 1% NaOH [46]. The influence of alkali concentration on polysaccharide extraction has also been investigated. For instance, Chen et al. [47] explored various NaOH concentrations for the extraction of polysaccharides from *Polygonatum odoratum*, revealing that the concentration of NaOH significantly impacts the extraction process and it is faster than hot water extraction [48].

**Figure 3:**
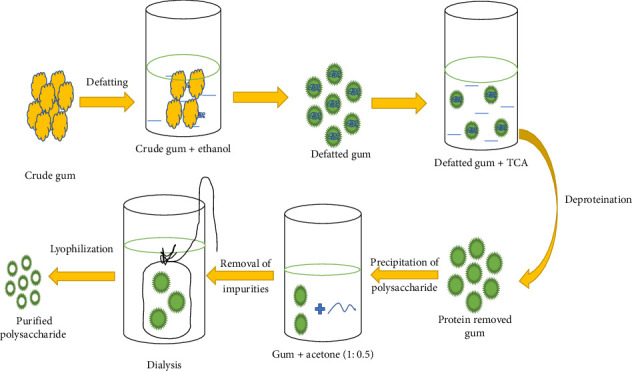
Extraction of plant gum polysaccharide.

In contrast to alkaline extraction, acid-based methods also play a role in polysaccharide isolation, as demonstrated in the *Crassostrea rivularis* study using citric acid [46]. The choice of extraction method, whether alkaline or acidic, is often tailored to the specific characteristics of the polysaccharide and the source material to optimize yield and purity. It is essential to note that for certain plant gum polysaccharides of *Araucaria heterophylla* and *Prosopis chilensis*, a different approach involving overnight soaking in ethanol, deproteination with trichloroacetic acid, acetone precipitation, and dialysis is employed (Figure 3) [49, 50]. This highlights the diversity of extraction strategies depending on the polysaccharide's properties and source.

**Figure 4:**
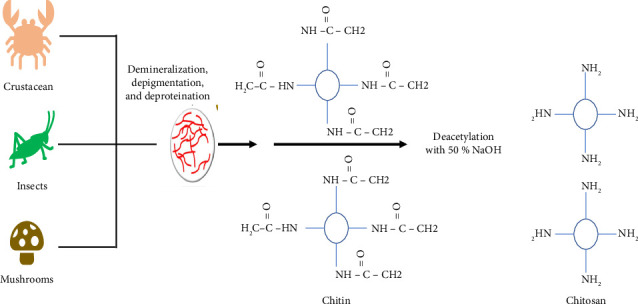
Purification of chitin from crustacean shells and conversion of chitin to chitosan.

The extraction of chitin and chitosan from crustacean sources commonly follows the processes that typically involve demineralization, deproteinization, and decolorization [51], followed by deacetylation to obtain chitosan (Figure 4). Although not directly focused on the acid/alkali solubility enhancement principle, these steps are crucial for the effective isolation of these important polysaccharides from their natural sources [52–55].

### 2.2. Physical Methods for Polysaccharides Extraction

#### 2.2.1. Ultrasonic-Assisted Extraction (UAE)

UAE stands out as a significant method for polysaccharide isolation, primarily due to its reliance on ultrasonic wave cavitation. This physical phenomenon effectively disrupts cell walls and accelerates the dissolution of intracellular components, leading to an enhanced polysaccharide yield [40]. The method is simple, remarkably stable under environmental stimuli, low energy consumption, and its efficiency even when operating under suboptimal conditions [40]. The significance of UAE is particularly evident when compared to traditional hot water extraction. Studies have shown that UAE can lead to a substantial increase in the yield ratio of glucose. This improvement is attributed to the cleavage of side chains containing *D*-mannose and *D*-galactose, with glucan typically forming the primary polysaccharide chain [56]. For instance, Aguiló-Aguayo et al. [57] successfully employed ultrasound-assisted extraction to isolate water-soluble polysaccharides from mushroom by-products. Their method involved mixing mushroom powder with distilled water, ultrasonication, and subsequent ethanol treatment, resulting in a significantly enhanced yield of polysaccharides from the fungal source [57]. However, it is crucial to acknowledge some notable drawbacks including the tendency for the UAE to yield impurities, often necessitating an additional purification step [58]. Furthermore, achieving a high extraction rate of polysaccharides using this method typically requires the application of increased ultrasonic power, which, in turn, demands elevated operating temperatures [59]. UAE has proven effective for the extraction of *Acacia seyal* gum (ASG), commonly known as Gum Arabic (GA). In this application, methanol was utilized as a solvent in conjunction with ultrasonication, not only aiding in gum extraction but also enhancing the extraction of polyphenols and antioxidant activities [60].

Beyond general polysaccharide extraction, UAE has also been applied to specific compounds like chitosan from North Atlantic Shrimps (*Pandalus borealis*) shells, where its influence on yield and morphology has been reported [61].

#### 2.2.2. Microwave-Assisted Extraction (MAE)

MAE stands out as an efficient method for isolating bioactive compounds, particularly polysaccharides. This technique leverages the direct interaction of electromagnetic waves with polar solvents, inducing rapid internal heating within the solvent. This localized and swift heating leads to the effective breakdown of cell walls, facilitating a faster and more comprehensive release of target compounds into the solvent [62]. A key advantage of this method lies in its ability to significantly reduce both extraction time and solvent consumption. By directly heating the solvent, MAE ensures improved yield and enhanced efficiency under precisely controlled conditions. This precise control over temperature and time is particularly beneficial for extracting heat-sensitive compounds, as it minimizes their degradation, thus preserving their bioactivity and integrity [62]. The type and volume of solvent, the applied temperature, extraction time, and solvent polarity used also influence the extraction [63, 64]. When extracting polysaccharides, careful consideration of the applied time and microwave power is essential to prevent their degradation [59]. The utility of MAE has been demonstrated across a range of polysaccharide extractions. For instance, Zhao et al. [65] employed MAE to extract polysaccharides from *P. ginseng*, optimizing parameters such as extraction time and temperature, microwave power, and the liquid-to-solid ratio to achieve efficient isolation. Similarly, Thirugnanasambandham et al. [66] successfully isolated polysaccharides from mulberry leaves using MAE. Their study highlighted the critical role of microwave power and extraction time, where mulberry powder dissolved in distilled water was subjected to varying times and powers, followed by ethanol precipitation.

Further research has also underscored the importance of specific MAE parameters. Zheng et al. [67] identified the liquid-to-solid ratio as a primary factor influencing the extraction of pumpkin polysaccharides, reporting a highest yield of approximately 16.76%, while time and temperature had a less significant impact. Another study by Ben Salem et al. [68] achieved a 2.76% polysaccharide yield from *Posidonia oceanica* (Neptune grass) using MAE with a power of 800 W, an extraction time of 60 s, and a liquid-to-solid ratio of 50:1 (mL/g). Beyond polysaccharides, MAE has also proven effective for extracting other biopolymers, such as chitin from *Rhizopus oryzae* NRRL 1526 biomass [69]. Its versatility extends to modifying biopolymers, as seen in the preparation of guar gum for superabsorbent hydrogel synthesis via MAE, yielding an impressive 89.2% grafting yield for GG-g-AAM4 [70].

#### 2.2.3. Infrared-Assisted Extraction (IR-AE)

IR-AE is an important technique for the purification of polysaccharides and holds significant potential for chitosan purification due to its innovative use of infrared radiation (750 nm–1 mm) to heat solvents, promoting cell disruption and the release of bioactive compounds [71]. This method offers substantial advantages over conventional approaches, including reduced cost, simplicity, user-friendliness, a radiation-free process, decreased extraction time, lower extraction temperatures, and higher extraction quotients [72]. For polysaccharides, IR-AE has proven highly effective, as demonstrated by Qu et al. [72] who used it to extract polysaccharides from *Bletilla striata* with subsequent optimization via response surface methodology (RSM) for temperature, extraction time, and water-to-solid ratio. Zhu et al. [73] found that polysaccharide extraction from coarse tea showed better results. The strong absorption characteristics of active compounds to IR wavelengths also make it more secure than MAE [74].

#### 2.2.4. Supercritical CO_2_ Extraction (SFE)

SFE, a pivotal “green” technology for the purification of polysaccharides, offers a superior alternative to traditional organic solvent methods by leveraging the tunable density and solvating power of supercritical carbon dioxide for selective component separation [71]. While CO_2_ is inherently nonpolar, the strategic addition of modifiers like ethanol and methanol is crucial for enhancing the solubility and extraction efficiency of more polar compounds, such as polysaccharides [48, 75]. For instance, Gong et al. [76] effectively extracted a high yield of polysaccharide (around 10 g) from *Ginkgo* leaves using ethanol as a cosolvent, optimizing the process with RSM at 63.0°C, 42 MPa, 1 h 39 min, and 68.0% ethanol. The application of SFE also extends to chitosan, as evidenced by Baldino et al. [77] who utilized SFE after dissolving chitosan in water and acetic acid. Furthermore, SFE has been successfully applied to extract valuable compounds like Chios mastic gum, known for its antimicrobial triterpenes against *Helicobacter pylori*, with Xynos et al. [78] reporting a significant yield of 198.2 g.

#### 2.2.5. Subcritical Water Extraction (SWE)

SWE, also known as pressurized hot water extraction, presents a highly promising and important method for polysaccharide purification due to its unique properties and demonstrated efficacy. This technique involves heating water above its boiling point (100°C) but below its critical temperature (374°C), while maintaining it in a liquid state under specific pressure [79]. The high temperatures (100−374°C) and pressures (> 50 bar) enable subcritical water to extract both polar and nonpolar compounds through minor pressure adjustments [80], making it a versatile solvent for diverse bioactive compounds. Its significance for polysaccharide purification is underscored by several studies: Zhang et al. [81] highlighted SWE as a more preferable, ecofriendly, and high-efficacy method for extracting polysaccharides from *Lentinus edodes*, a nutrient-rich edible fungus known for its polysaccharides, purines, and choline with antitumor, antiviral, and antimicrobial activities. Furthermore, Liu et al. [19] successfully isolated a significant 21.88% of polysaccharide from *Dendrobium nobile Lindl* using this method. The efficacy of SWE extends beyond direct polysaccharide extraction to their precursors and derivatives, as shown by Hao et al. [82], who efficiently prepared chitin from swimming crab (*Portunus trituberculatus*) shells and subsequently extracted chitin and chitosan using SWE. Their work impressively demonstrated that subcritical water pressurized at 100−374°C could promote α-chitin decomposition within just 7 min, followed by enzymatic degradation. Crucially, the subcritical water pretreatment drastically reduced the element-chelating ability of chitin, thereby facilitating easier demineralization [82].

#### 2.2.6. Dynamic High-Pressure Microjet Technology (DHPM)

DHPM is a method for polysaccharide purification, leveraging high-frequency vibration, shear, instantaneous pressure drops, and cavitation at pressures up to 200 MPa to efficiently disrupt cells and release biopolymers by forcing samples through a microsized nozzle [83]. This technique's significance lies in its ability to achieve high extraction rates and significantly reduce extraction time, while also enhancing the solubility of biopolymers by reducing particle size and requiring minimal solvent use [83, 84]. Li et al. [85]demonstrated the potential of DHPM method which improved solubility, emulsifying capacity and stability. This technique is a green processing technology which enhances the functional properties of biopolymers with minimal solvent use. However, a notable drawback is the potential for polysaccharide chain breakage and alterations to their original characteristics due to the intense forces involved [84], necessitating careful parameter optimization to balance efficiency with structural integrity.

### 2.3. Biological Methods for Polysaccharide Extraction

#### 2.3.1. Enzymatic Hydrolysis

Enzymatic hydrolysis is a highly valuable and important technique for polysaccharide purification within the context of biomass saccharification, effectively breaking down complex polysaccharides into simpler monosaccharides [86]. This method is widely recognized for its ecofriendly nature and ease of operation, making it a prominent choice for natural product extraction. A key advantage is its effectiveness at lower temperatures and shorter durations, which crucially prevents structural damage to polysaccharides and avoids the release of unwanted cellular components [46, 87, 88]. For instance, a study demonstrated that enzyme-assisted extraction was the most efficient method for obtaining polysaccharides from coarse green tea [73]. Successful implementation of this technique necessitates careful consideration of critical parameters such as enzyme concentration, pH levels, and extraction temperature [88]. Its versatility is highlighted by Bhotmange et al. [87], who used trypsin, pectinase, and papain enzymes for the isolation of polysaccharides from *Tuber aestivum* (summer truffle), followed by 80% ethanol precipitation. Similarly, Lukova et al. [89] utilized hemicellulase and mannanase enzymes to extract polysaccharides from *Plantago major L.* leaves, specifically targeting xylan and galactomannan substrates, concluding that hemicellulase was particularly effective for this species. While the text primarily focuses on polysaccharide extraction from plant sources, the references to chitin preparation from shrimp shells by Qin et al. [90].Hosney et al. [91]have detailed about involvement of heating and centrifugation along with enzymatic methods for purification of animal derived polysaccharides.  Table 1 briefs the advantages and disadvantages of different extraction methods.

## 3. Purification of Polysaccharides

**Figure 5:**
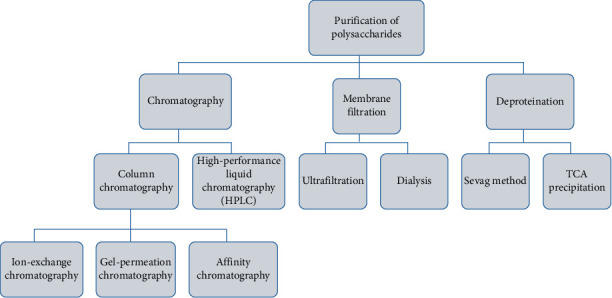
Different approaches in the purification of polysaccharides.

Once the extraction process is completed, the immediate next step is separating the extract from the impurities present, leading to the formation of a composition which primarily consists of the polysaccharides. The newly formed mixture of polymerized components represents a complex array of molecules that need to undergo fractionation and depolymerization to facilitate a detailed examination of their biological functions and intricate structure. Various purification techniques are employed in isolating and refining the polysaccharide components, and some of them include precipitation, gel chromatography for size-based separation, anion exchange chromatography for charge-based separation, macroporous resin column chromatography for specialized purification needs, ultrafiltration for fine particle separation, and an array of other similar methodologies. These intricate procedures play a crucial role in preparing the polysaccharide samples for further analysis and study, ensuring that the extracted components are free from unwanted substances and uniformly processed for accurate experimentation and investigation into their biological properties. Different approaches used in the purification of polysaccharides are listed in Figure 5. The polysaccharides which are obtained by the extraction, separation, and purification techniques yield more homogeneous polysaccharide fractions for characterization [94]. The methods for polysaccharides purification start with the removal of impurities and are further purified by physical or chemical methods. Each purification method has its own merits and demerits, which is listed in Table 2.

### 3.1. Deproteination of Polysaccharides

The removal of proteins is a critical step in the separation and purification of polysaccharides due to the latter's hydrophilic nature, which often leads to the formation of protein–polysaccharide complexes. One widely utilized deproteination technique is the Sevag method, which effectively denatures proteins without typically affecting the polysaccharides themselves [95, 96]. This method was notably employed for crude polysaccharide solutions from *Lycium barbarum* berries [97]. However, a significant limitation of the Sevag method is that some studies suggest it may lead to a loss of polysaccharides [96]. As an alternative, Zhang et al. [98] explored the trichloroacetic acid (TCA)/acetone precipitation method, which not only deproteinates but also allows for the isolation of polysaccharides. Another chemical approach, the use of hydrochloric acid, is mentioned, but it carries the drawback of potentially causing partial hydrolysis of polysaccharides, thereby affecting their preservation [98]. Beyond these, the three-phase partitioning (TPP) protein separation method, which integrates salting out, isoelectric point precipitation, and solvent precipitation, has been successfully applied to clear protein impurities from polysaccharides extracted from *C. fluminea* (golden clam), proving its effectiveness in purification [99]. Furthermore, the ammonium sulfate salting-out method alone is considered a more delicate approach, capable of removing protein from protein–polysaccharide complexes without harming their biological properties [96]. For specific cases like chitosan, enzymatic deproteination has also been reported, such as the use of immobilized pepsin to treat chitosan suspensions, followed by neutralization, filtration, and lyophilization to obtain purified chitosan [95]. The diverse array of methods highlights the importance of selecting an appropriate deproteination technique, weighing its efficiency against potential polysaccharide loss or degradation, to ensure optimal purity and preservation of the target biopolymer.

#### 3.1.1. Anion Exchange Column Chromatography

Anion exchange chromatography is an important and widely used method for polysaccharide purification, primarily due to its ability to differentiate polysaccharides based on their distinct ionic properties, including acidic, neutral, and viscous types, as well as those bound to proteins [100]. The fundamental principle of this separation relies not only on ion-exchange but also crucially on electrostatic interactions between charged polysaccharides and the column matrix. Typically, at a pH of 6.0, acidic polysaccharides are effectively adsorbed onto the exchanger, while neutral polysaccharides do not exhibit this adhesion [101]. This differential binding allows for selective separation. Prominent examples of its application include the purification of water-soluble polysaccharides from *L. lucidum* (Chinese privet) using DEAE-52 cellulose gel, followed by elution with varying concentrations of NaCl [102]. Similarly, the purification of *G. lemaneiformis* (Red alga) polysaccharides was successfully achieved by applying them onto a DEAE Sephadex A-50 column and subsequently eluting with a combination of distilled water and NaCl solution [103]. Despite the generally favorable separation results, a notable limitation of this technique is its sensitivity to changes in elution rate due to fluctuations in volume, pH, or variations in the ionic strength of the solution [103], which can complicate method reproducibility and require precise control during the purification process.

#### 3.1.2. Gel Column Chromatography

Gel column chromatography stands as a cornerstone in polysaccharide purification, recognized as one of the most effective techniques for separating these complex biopolymers based on their distinct sizes and shapes, a process aptly termed the sieve principle [40]. Its widespread utilization highlights its importance in refining crude polysaccharides, making it an indispensable tool in the field. The method relies on a stationary phase composed of gels such as Sephadex, agarose, and dextran, which are critical for the effective elimination of impurities, including small molecules and inorganic compounds. Concurrently, diluted salt solutions and deionized water serve as essential eluents, facilitating the separation process [104]. Despite its high efficacy in separating polysaccharides by size, a significant limitation of gel column chromatography is its relatively low resolution for polysaccharides of similar molecular weights, which can lead to incomplete separation or overlapping peaks. Additionally, the technique can be time-consuming and may require large volumes of eluents, particularly for large-scale purifications, which can also be a cost consideration.

#### 3.1.3. Affinity Column Chromatography

This method, based on molecular affinity, is an effective technique for purifying a variety of biocompounds, with particular importance for polysaccharide purification, especially those characterized by noncovalent interactions with ligands [105]. Its fundamental principle relies on employing a covalently bound matrix ligand that uniquely bonds with diverse target ligands possessing a range of functional groups, thereby ensuring successful purification [105]. However, a primary challenge and major drawback of this approach is the identification of suitable ligands for effective binding. Despite this limitation, optimizing the specific chromatographic column can effectively overcome this hurdle, enabling the purification of specific polysaccharides that exhibit a strong affinity toward that particular column [40].

### 3.2. Other Purification Techniques

The purification of polysaccharides is a complex and crucial process, with the size and shape of these biopolymers being key determinants in selecting the most suitable purification method and influencing the rate of purification [101, 106]. Various techniques are employed, each leveraging different principles: dialysis and ultrafiltration depend on molecular size, utilizing semi-permeable membranes, while centrifugation separates based on both size and density through varying speeds [101, 106]. For example, in *Lycium barbarum*, polysaccharides with anticancer potential were fractionated by high-performance size exclusion chromatography (HPSEC) and further analyzed for their monosaccharide composition by gas chromatography, highlighting the importance of combining purification with structural characterization techniques [107]. Despite the availability of these methods, a significant limitation remains that obtaining purely isolated polysaccharides is challenging due to the absence of a universally accepted, foolproof methodology, which further complicates achieving complete purification given the intrinsic structural complexities of polysaccharides [40]. Consequently, current methodologies often fall short in ensuring the full isolation of polysaccharides, underscoring the critical need for technological advancements to address these gaps and, importantly, to preserve the structural integrity and biological properties of the isolated polysaccharides [40]. Table 2 briefs the various purification methods along with their advantages and disadvantages.

## 4. Characterization of Polysaccharides

Once extraction and purification of the product of interest are completed, before finding their applications, characterizing the product is crucial in order to have a thorough understanding of its biological properties, physicochemical properties, structure, surface properties, etc., using various analytical approaches. When it comes to the fabrication of natural biopolymers, it is inevitable to have detailed knowledge about their structural, surface, biological, nontoxicity, and immunological properties. For any naturally derived polysaccharide, the most commonly done analytical characterization techniques are UV-Visible (UV–Vis) spectroscopy, Fourier transform infrared spectroscopy (FTIR), x-ray diffraction (XRD) analysis, thermogravimetric analysis (TGA), scanning electron microscopy (SEM), transmission electron microscopy (TEM), gas chromatography–mass spectroscopy (GC–MS), and nuclear magnetic resonance (NMR) [108, 109]. The various characterization techniques are discussed below.

### 4.1. UV–Vis Spectroscopy

UV–Vis spectroscopy is a versatile and nondestructive analytical technique that measures the absorbance or transmittance of light in the 200–800 nm range, encompassing both ultraviolet and visible spectra [110, 111]. This technique is invaluable in polysaccharide research as it allows for the study of electronic transitions and the detection of modifications involving chromophoric functional groups. For instance, the presence of specific absorption bands can indicate the incorporation of other materials or structural changes. Dima et al. [112] utilized UV–Vis analysis (200–1000 nm) on chitosan–nanolignin aerogels to identify sulfides with *n *⟶*  σ*^∗^ transition bands at characteristic wavelengths (e.g., 210 nm for (CH_3_)_2_S, 215 nm for (C_2_H_5_)2S, 235–240 nm for R2S, and 250 nm for RSSR disulfides), concluding that chitosan biopolymers effectively retain carbon nanoparticles in sulfite liquor, and thus, UV–Vis can confirm the presence of specific chemical species and their interactions within polysaccharide composites.

Furthermore, UV–Vis spectroscopy is crucial for characterizing the optical properties of polysaccharide-based materials and their potential applications. M'sakni & Alsufyani [113] analyzed cellulose and starch biocomposite films containing green silver nanoparticles for food packaging and optoelectronic devices, observing a maximum absorbance at 415 nm, which shifted to 432 nm with increased incubation time. This peak shift was attributed to surface plasmon resonance of electrons on the nanoparticles, indicating an increase in silver nanoparticle size. Similarly, Chia et al. [114] fabricated starch/polyaniline-based biopolymer films for food packaging and ammonia sensing. Their UV–Vis spectrometry (250–900 nm) revealed peaks at 376 nm (π–π∗ transition in the benzenoid ring) and 457 nm (polaron–π∗ transition in the quinoid ring), confirming the polyaniline's emeraldine salt form. Bassi et al. [115] also utilized UV–Vis to analyze copper oxide nanorods prepared with agar and alginate biopolymer matrices, finding an absorption peak at 271 nm and a blue shift in CuO, suggesting successful functionalization of nanoparticles within the biopolymer matrix. Thus, UV–Vis analyzes the behavior within biopolymer matrices and validates the desired chemical states for specific applications.

Beyond material characterization, UV–Vis spectroscopy plays a vital role in assessing the purity of polysaccharide extracts by detecting common contaminants. Jiang et al. [116] confirmed the absence of protein and DNA in *Plumula nelumbinis* polysaccharides (LNP I, II, and III) by the lack of peaks at 260 nm and 280 nm, respectively. Likewise, Cai et al. [117] also utilized UV–Vis to verify the absence of nucleic acid/protein in polysaccharides extracted from American ginseng and green tea, respectively, although Cai et al. [117] did find contaminants in one sample. Liao et al. [118] employed UV–Vis (190–900 nm) to determine the structural composition of polysaccharides from mulberry leaves, observing peaks between 190 nm and 215 nm for different solvents, indicating the presence of polysaccharides and their diverse structural compositions, while also confirming the absence of protein or nucleic acid. Conversely, Wang et al. [119] characterized polysaccharides from *Cortex Periplocae*, and despite purification steps like decoloration and deproteinization, their UV–Vis analysis revealed peaks around 240 nm, confirming the presence of residual protein and nucleic acid in the crude and purified polysaccharide fractions (CPP1, CPP2, and CPP3).

### 4.2. FTIR

FTIR spectroscopy is a rapid analytical tool for the comprehensive characterization of polysaccharide samples, primarily by determining their functional groups and identifying specific polysaccharide types through unique spectral signatures [116]. The technique relies on the identification of characteristic peaks corresponding to specific molecular vibrations. For instance, the presence of O-H groups, a hallmark of hydroxyl functionalities common in polysaccharides, is indicated by peaks within the 3550–3220 cm^−1^ range. Similarly, C=O groups, characteristic of carbonyl functionalities, are identified by peaks around 1650 cm^−1^, while C-H stretching vibrations, indicative of alkane or other carbon–hydrogen bonds, appear around 2900 cm^−1^ [58]. The ability to discern these distinct spectral features allows researchers to accurately determine the composition of various polysaccharides, as each is defined by a unique combination of functional groups [120]. Studying these functional group using FTIR allows researchers to confirm the presence of target polysaccharides and monitor changes in their structure during purification or modification processes, which is crucial for controlling product quality and functionality.

Recent research has extensively utilized FTIR to explore diverse applications of polysaccharides, demonstrating its capability to identify and characterize molecular interactions within complex polysaccharide systems. In drug delivery, Almukainzi et al. [121] used FTIR to analyze chitosan/hesperidin nanoparticles, identifying amine I NH-H bending (1600 cm^−1^), amide II carbonyl (1670 cm^−1^), *p*=O interactions, OH stretching (2923.09 cm^−1^), carbonyl groups (1644.35 cm^−1^), and aromatic rings (1517.22 cm^−1^). For edible films, Castro et al. [122] employed FTIR to analyze ginger essential oil (GEO)-loaded bentonite clay particles within chitosan–cassava starch films, identifying starch structures (1010–1100 cm^−1^), chitosan amino groups (1519–1529 cm^−1^), O–H and N–H oscillations (3000–3600 cm^−1^), and C–H stretching (2850–2960 cm^−1^, 1377 cm^−1^). Furthermore, Davis et al. [123] utilized FTIR to confirm functional groups in fungal chitosan from pineapple peel, observing O–H and N–H stretching (3550–3096 cm^−1^), C–H stretching (2921.73 and 2850.33 cm^−1^), N–H bending (1638.11 cm^−1^), and C–O stretching (1022.46 cm^−1^). Thus, FTIR allows researchers to focus on specific interactions crucial for the design and performance of polysaccharide-based materials in various industries. Moreover, FTIR has proven invaluable in tissue engineering and biopolymer research, providing critical insights into the structural and functional properties of polysaccharides across a wide spectrum of scientific and industrial applications. Lopes et al. [124] analyzed chitosan, sodium alginate, and calcium phosphate scaffolds, observing strong bands indicating hydrogen bond formation (3500–3100 cm^−1^) and C=N bonds for crosslinking, which are vital for understanding scaffold integrity. Tritean et al. [125] studied biopolymeric hydrogels with selenium nanoparticles, identifying amide bands (1600 cm^−1^), alcohol bands (1030 cm^−1^), C–N bands (1070 cm^−1^), and C–H bands (2918 and 2849 cm^−1^), which are indicative of the polymer network. Researchers have also used FTIR for detailed structural elucidation of polysaccharides from various natural sources: Zhang et al. [126] noted pyranose rings (1050 cm^−1^), mannose content (810 cm^−1^), and O–H stretching (3410 cm^−1^) in *Umbilicaria esculenta*; Shi et al. [103] identified hydroxyl stretching (3400 cm^−1^), glycosidic linkages (1072 cm^−1^), and sulfate groups (1260 cm^−1^) in red algae polysaccharides; and Huang et al. [85] and Chen et al. [58] similarly used FTIR to characterize tea and *Ginkgo biloba* polysaccharides, respectively. The analysis of gum derived from *Albizia zygia* by Eddy et al. [127] revealed C–H bending, C–O stretching, C–N stretching, and N–H bending vibrations within the 400 to 4000 cm^−1^ range. Similarly, Udo et al. [128] performed physicochemical and FTIR studies on gums from *Acacia senegal* and *Anacardium occidentale*, observing a range of vibrations including C–C bending, CO_2_ bending, C–O stretch, C–H wagging, C–H in-plane, C=C stretching, C–H stretching, C=O asymmetric stretch, and O–H stretch in the *Acacia senegal* gum and similar bands in the *Anacardium occidentale* gum. Accordingly, FTIR enables researchers to pinpoint specific structural features that influence the biological activity and physical properties of polysaccharides, thereby allowing a focused approach to developing novel applications in areas like biomaterials and functional foods.  Table 3 comprises the different types of polysaccharides and their functional groups identified using FTIR, and their peak positions given by the analysis.

### 4.3. XRD Analysis

XRD is an indispensable technique for characterizing polysaccharides, offering critical insights into their crystalline nature by analyzing atomic and molecular lattice arrangements and diffraction patterns within crystal structures [109, 133]. XRD analysis enables researchers to determine angular diffraction patterns (2*θ*) and to identify crystal phase compositions, and quantify crystallinity indices. The versatility of XRD in characterizing polysaccharides is evident in various applications. For instance, the analysis of fungal chitosan extracted from pineapple peel revealed characteristic 2*θ* values at 12.33° (amorphous phase) and 22.66° (crystalline phase), with a crystallinity index of 51.61%, highlighting its potential as a sustainable plastic alternative [123]. Similarly, the examination of clay used in chitosan composite beads identified peaks indicative of illite, quartz, and kaolinite, confirming successful extraction of kaolinite [134]. Furthermore, XRD studies on chitosan-based mixed-matrix scaffolds for tissue engineering showed semicrystalline sodium alginate peaks and determined the crystalline size of hydroxyapatite, suggesting potential applications in bone repair [124].

The detailed structural information provided by XRD directly contributes to the development of polysaccharides for diverse applications. In antimicrobial fabric coatings, XRD confirmed the hexagonal crystal structure of zinc oxide particles in chitosan-coated cotton fabric [135]. For wound healing, XRD analysis of PVA nanofibrous mats containing chitosan, graphene oxide (GO), and carbon quantum dot-doped TiO2 (CQD-TiO2) confirmed proper crosslinking by identifying characteristic peaks for GO and CQD-TiO2 [136]. Agricultural applications also benefit from XRD, as demonstrated by the analysis of mancozeb-loaded chitosan–gum acacia nanocomposites, where highly crystalline mancozeb peaks and a broad chitosan peak confirmed successful mancozeb encapsulation [137]. The varying degrees of crystallinity are crucial for function; for example, extracellular polysaccharide (EPS) produced by *B. pseudomycoides* U10 exhibited poor crystallization [138], while a comparative study showed chitin to be the most crystalline and chitosan the least, with distinct peaks for cellulose, chitin, and chitosan [108]. The semicrystalline nature of bletilla rhizome polysaccharides (RBPs) with diffusion peaks around 20.4°, and crystalline states for RBP60 and RBP70, demonstrates temperature-dependent aggregation structures, which can be tailored for specific uses [139].

Beyond the applications mentioned, XRD is fundamental in understanding the foundational properties that dictate polysaccharide utility. Govindan et al. [140] utilized XRD to analyze the structural properties of chitosan–silver nanoparticles, determining a simple cubic crystal structure from peaks at 11.7° and 20.2° [140]. This structural understanding is vital for controlling nanoparticle behavior. Gartner et al. [141] correlated structure and dynamics in chitosan films, revealing that chitosan powder and neutralized films exhibit more amorphous structures compared to chitin, while highly acetylated chitosan showed five crystalline reflections, suggesting better organized long-range structures that could influence film performance [141]. Furthermore, the characterization of novel plant gums also relies heavily on XRD. For instance, a plant gum from *Cissus rufescens* showed strong peaks at approximately 14°, 15°, 24°, and 26° 2*θ*, with weaker peaks at 16° and 23° 2*θ*, indicating both amorphous and crystalline portions [142]. In contrast, the gum derived from *Cochlospermum gossypium* showed no significant peaks, suggesting a completely amorphous nature [143]. These findings underscore how XRD's ability to delineate crystallinity and structural details is paramount in tailoring polysaccharides for a vast array of material science, biomedical, and agricultural applications.

### 4.4. TGA

TGA is a powerful analytical technique for characterizing biopolymers by monitoring mass changes as a function of temperature. Utilizing a sophisticated instrument equipped with an oven, temperature programmer, sensitive balance, and atmospheric control, TGA precisely determines volatile components and moisture content, particularly in polymers. While most polysaccharides typically decompose below 200°C, some polymers exhibit remarkable thermal stability, enduring temperatures up to 300°C in air or 500°C in inert gas without structural or strength compromise. This capability is crucial for understanding the intrinsic thermal properties of polysaccharides and their suitability for various applications. For instance, TGA was effectively used to analyze the thermal stability of crude polysaccharides from *Solen marginatus* flesh, allowing for the determination of decomposition temperatures and a comparison of stability between crude and deproteinized fractions [144]. Similarly, the characterization of natural gums from *Diospyros melanoxylon*, *Buchanania lanzan*, and *Manilkara zapota* seeds through TGA and differential thermal analysis (DTA) revealed distinct mass loss events corresponding to moisture desorption and polysaccharide decomposition [145]. Furthermore, TGA confirmed the thermal protection offered by gray fruit extract encapsulated in microparticles, observing characteristic mass losses at approximately 100°C (water loss) and 303°C (saccharide degradation) [146].

The versatility of TGA extends significantly to evaluating the thermal stability of various biopolymer-based materials, directly impacting their potential applications. For instance, TGA was instrumental in assessing the thermal properties of chitosan-based films intended for food packaging, with mass loss near 450°C indicating high thermal stability and promising prospects for the food industry [147]. In the development of biomaterials, TGA insights into polyvinyl alcohol–chitosan scaffolds with silicon dioxide nanoparticles revealed distinct mass loss stages corresponding to water evaporation (100°C–150°C), PVA side chain disintegration and chitosan decomposition (200°C–400°C), and chitosan glycosidic bond degradation (400°C–480°C) [148]. This detailed thermal breakdown information is vital for optimizing scaffold design. Similarly, TGA performed on mancozeb-loaded chitosan-gum acacia nanocomposites showed a two-stage mancozeb particle breakdown and mass loss, confirming enhanced thermal stability in the nanoform, which is critical for controlled release applications [137]. Further studies on insulin release from chitosan hydrogels using TGA revealed initial water evaporation at 150°C and thermal degradation at 270°C, with grafting improving stability up to 600°C, demonstrating how thermal modifications can expand application horizons [149]. TGA also proves useful in analyzing complex green polymer mixtures from pine needles and phenolic matrices [150].

TGA is frequently coupled with other thermo-analytical techniques like differential scanning calorimetry (DSC) and gas-phase analytical methods such as FTIR, GC, and MS to provide a more comprehensive understanding of complex materials. This powerful combination enhances the ability to interpret and evaluate thermal behavior, allowing for quantitative component evaluation, chemical stability determination, and identification of environmental effects [151].

### 4.5. SEM

SEM is an indispensable tool for characterizing the surface morphology and shape of polysaccharides, offering detailed insights beyond the capabilities of optical microscopy. Unlike optical microscopes, which use light, SEM utilizes electron beams, allowing for the analysis of backscattered electrons (composition and topography), diffracted backscatter electrons (crystalline structures), and X-rays (elemental composition). While SEM excels at surface characterization, it does not provide information about internal structures [152]. This technique enables researchers to visualize intricate surface features and structural variations resulting from different processing methods or compositional differences, which is crucial for understanding and optimizing their functional properties.

Numerous studies highlight the diverse applications of SEM in polysaccharide research by correlating their morphology with potential uses. Wang and Guo [153] used SEM to analyze *Auricularia cornea* polysaccharides, revealing that different extraction and purification methods resulted in distinct microstructures. Dialyzed polysaccharides exhibited entangled structures with small particles, while purified fractions like ACPN-1a and ACPA-2a showed more uniform surfaces. ACPA-1a, a neutral polysaccharide, displayed fibrous filaments and chain conformations, whereas acidic polysaccharides showed fewer filaments and more particles, indicating how processing influences their physical forms [153]. Reyna-Urrutia et al. [146] employed SEM to examine microparticles fabricated with gray fruit extract, observing a heterogeneous morphology with irregularities and round bodies indicative of a 3D encapsulation network, suggesting their suitability for controlled release applications [146]. Almukainzi et al. [121] utilized SEM to characterize chitosan/hesperidin nanoparticles, revealing irregular, rough surfaces in hesperidin particles and spherical nanoparticles under 40 nm, with crosslinking observed at lower magnification, which is vital for understanding drug delivery systems [121]. Similarly, Lopes et al. [124] investigated chitosan-based scaffolds, observing interconnected pores ranging from 5 to 150 μm, demonstrating how scaffold formulation influenced pore size, a critical factor for tissue engineering applications [124].

Furthermore, SEM has been instrumental in evaluating polysaccharide structures in diverse contexts, allowing researchers to correlate structural features with functional properties. Maćczak et al. [154] used SEM to analyze chitosan bioflocculants, observing flake-shaped structures with lengths of 50–300 μm, along with granular groupings and spindle structures that contribute to flocculation potential, highlighting their use in water treatment [154]. Luca et al. [155] confirmed the microporous morphology of methacrylated biopolymer hydrogels using SEM, crucial for controlled drug release, emphasizing their biomedical applications [155]. Hu et al. [156] utilized SEM to characterize *Pinelliae Rhizoma Praeparatum Cum Alumine* polysaccharides, revealing differences in surface morphology between acidic and neutral fractions, with TPN-II showing a rough, uneven surface and TPA-II a homogeneous, loose structure, indicating how structural variations can impact their biological activities [156]. Sugiyanti et al. [157] extracted chitosan from shrimp and crab shell waste and found it to be smooth and fine morphology using SEM. Savi et al. [158] employed SEM to compare dialyzed and nondialyzed polysaccharides from *Dioscorea bulbifera*, observing that the nondialyzed polysaccharide was more compact and densely packed, influencing their potential as food additives or pharmaceutical excipients [158].

The insights gained from SEM analysis are crucial for tailoring polysaccharides for specific uses. Amaral et al. [159] studied the structural and functional properties of chitosan after chemical modifications mediated by phosphorylation, with SEM images of the unmodified, cross-sectioned, and phosphorylated chitosan membranes revealing a smooth and homogeneous film surface without any blisters or bubbles postchemical treatment. This smooth morphology is desirable for various membrane applications [159]. Arafat et al. [160] extracted chitosan from waste shrimp shells and characterized it, with SEM images demonstrating nonhomogeneous and nonsmooth surfaces that helped in analyzing their surface morphology and potential applications where surface roughness is a factor [160]. Agarwal et al. [161] synthesized and characterized chitosan nanoparticles, performing SEM analysis to determine their surface morphology and shape. The results showed that the synthesized chitosan nanoparticles have a very homogeneous morphology and spherical shape, with a size of about 80–100 nm, which is ideal for drug delivery or nanomedicine [161]. Eddy et al. [127] studied the physiochemical and rheological properties of the gum extracted from *Albizia zygia*, with SEM images showing the polymers seemed to accumulate in the hair fiber of the tree and the morphology was irregular in shape, impacting its potential in cosmetic or pharmaceutical formulations [127].

### 4.6. TEM

TEM stands as a powerful technique for characterizing polysaccharides, offering unparalleled insights into their morphology, composition, and crystallographic structure. Distinguishing itself from SEM by detecting transmitted electrons, TEM achieves significantly higher magnification and resolution, allowing for the visualization of internal sample compositions and intricate nanoscale details in high-resolution, two-dimensional black-and-white images [162]. Modern TEM instruments have further advanced, providing both high spatial and energy resolution, which is critical for comprehensively characterizing nanomaterials in terms of size, phase, composition, morphology, and strain [163]. This detailed characterization capability is fundamental to understanding how polysaccharide structures can be engineered for diverse applications.

In polysaccharide research, TEM has proven instrumental in visualizing nanoparticle structures and arrangements, directly impacting their functional design. For instance, the characterization of nanoparticles of fucoidan (FC), CS, and chitosan via TEM revealed agglomeration effects and the presence of non-nanostructured material sizes, which is crucial for predicting their in vivo coacervation behavior [164]. Understanding the microstructure of purified polysaccharide JUYP from *Umbilicaria yunnana* through TEM, which showed a main linear chain with side chains forming a “bow tie” structure, directly contributed to elucidating its bioactivities [165]. Similarly, in drug delivery applications, TEM confirmed the successful formation of cross-linked spherical chitosan/hesperidin nanoparticles with specific size ranges [121] and observed quasispherical selenium nanoparticles within a biopolymeric hydrogel, accurately determining their dimensions [125]. These precise morphological details are essential for optimizing drug encapsulation and release.

The utility of TEM extends further into engineering polysaccharide-based materials for specific functionalities. For instance, the visualization of chitosan-coated halloysite nanotubes for controlled drug release revealed hollow tubular nanoparticles with uniform chitosan coating, confirming the successful development of a controlled release system [166]. Furthermore, TEM was used to characterize polysaccharide-coated iron oxide nanoparticles (IONs) produced by *Staphylococcus warneri*, identifying spherical IONs with a 34 nm diameter without the need for staining, and enabling the calculation of average cone diameter and particle size distribution [167]. This level of detail is vital for tailoring nanoparticles for biomedical imaging or targeted therapies. In another application, the green synthesis of copper–chitosan nanoparticles was confirmed by TEM images, which showed spherical particles sized 20–30 nm and demonstrated the stability of copper nanoparticles in the presence of chitosan, preventing agglomeration or oxidation [168]. Moreover, TEM analysis of gum polysaccharide extracted from *Cochlospermum gossypium* DC revealed an extending backbone chain with an extensively branched structure for the native gum, while the deacetylated form showed randomly shaped large worm-like structures [169]. These insights into molecular architecture are paramount for understanding and optimizing the beneficial properties of such gum polysaccharides in various fields. Collectively, these studies underscore TEM's crucial role in elucidating the structural properties of polysaccharide-based materials at the nanoscale, directly facilitating advancements in their diverse applications.

### 4.7. GC–MS

GC–MS stands as a pivotal and highly advanced analytical technique in chemistry, enabling precise identification, quantification, and comprehensive profiling of compounds, whether targeted or untargeted. The methodology involves an initial vaporization of the sample, which then traverses a chromatographic column. Within this column, different compounds separate based on their distinct chemical properties, such as boiling point and polarity, causing them to elute at varying speeds. Upon exiting the GC system, these separated compounds enter the MS component, where they are ionized, fragmented, and subsequently separated according to their mass-to-charge ratio. This powerful combination proves invaluable in biopolymer research for the identification and characterization of monomers and degradation products that arise from polymer breakdown. A prime example of its efficiency is demonstrated by Khang et al. [170], who utilized pyrolysis–gas chromatography–mass spectrometry (Py–GC–MS) to determine the monomer composition in microbial cells in a significantly shorter duration and without requiring extensive pretreatment steps.

The insights gained from GC–MS analyses are crucial for understanding the stability and potential applications of polysaccharides. For instance, Zeng et al. [171] employed GC–MS to investigate the volatile chemicals produced during the pyrolysis of chitin, successfully identifying compounds such as acetamide, furfuryl alcohol, 2-pyrrolealdehyde, 3-acetamidofuran, and 2-furfural. Such detailed identification of degradation products is vital for predicting material behavior under thermal stress and for designing processes to manage or utilize these volatile compounds. Similarly, the technique was instrumental in investigating the thermal degradation kinetics and pyrolysis characteristics of a complex chitosan–Zn matrix. This analysis revealed a complex pyrolysis mechanism leading to the production of numerous volatile compounds, providing critical data for optimizing material processing and understanding its environmental impact or potential for new applications based on its degradation profile [172].

Furthermore, GC–MS plays a significant role in characterizing the compositional richness of natural polysaccharide sources, directly informing their potential utility across various industries. Eddy et al. [127] leveraged GC–MS studies to reveal the presence of several important and beneficial compounds, including (E)-methyl octadec-7-enoate, methyl palmitate, and methyl stearate, within the gum derived from *Albizia zygia*. The identification of such beneficial compounds suggests potential applications for this gum in pharmaceuticals, cosmetics, or food additives. In a similar vein, researchers evaluated the gum obtained from *Cochlospermum gossypium* using GC–MS, which facilitated the identification of various neutral sugars and uronic acids, such as rhamnose, galacturonic acid, glucuronic acid, β-*D*-galactopyranose, and α-*D*-glucose [143]. The precise knowledge of these monomeric units is fundamental for understanding the gum's functional properties, such as its gelling, emulsifying, or thickening capabilities, thereby guiding its application in food, pharmaceutical, or industrial formulations.

### 4.8. NMR Spectroscopy

NMR is a powerful analytical technique widely employed for determining the structure, properties, and dynamics of molecules within a sample. Beyond providing fundamental insights into molecular composition, NMR spectra offer crucial information regarding the connectivity and arrangement of atoms, which is essential for identifying unknown compounds. Furthermore, NMR allows for the study of different functional groups present in a sample and their interactions [173]. This comprehensive structural elucidation capability makes NMR an invaluable tool for characterizing polysaccharides, enabling a deeper understanding of their properties and potential applications.

The precise structural details revealed by NMR are critical for understanding the functional properties of polysaccharide gums. For instance, Vinod et al. [143] utilized NMR to examine the physicochemical and structural properties of gum extracted from *Cochlospermum gossypium*. Their ${ }^{13}$C and proton NMR spectra unveiled a complex arrangement of sugar residues, including *α*(1-2) β-*D*-Galp, (1-6)-β-*D*-Galp, (1-4) β-*D*-GlcpA, 4-*O*-Me-α-*D*-GlcpA, and (1-2) α-L-Rha [143]. Similarly, Gutiérrez de et al. [174] analyzed the polysaccharide gum from *Spondias purpurea* var. *lutea* using 1-D and 2-D NMR spectroscopy. By studying both the original gum and its degradation products, they successfully elucidated the complete structure, revealing its composition of 3-O- and 6-O-galactosyl residues, terminal and 3-*O*-α-*L*-arabinofuranosyl, terminal rhamnosyl residues, and uronic acids [174]. These detailed structural insights are fundamental for predicting and controlling the behavior of these natural polymers in various applications, such as their use as thickeners, emulsifiers, or stabilizers in the food and pharmaceutical industries.

Further demonstrating its utility, NMR provides critical data for the characterization and comparison of polysaccharide extracts. Saeidy et al. [175] employed 1D and 2D NMR spectra to study the structural properties and thermal behavior of gum obtained from *Ferula assa foetida L.*, with ^1^H NMR and ^13^C NMR yielding signals related to anomeric carbons, and 2D NMR providing information on overlapped peaks and correlations [175]. This level of detail is crucial for quality control and for optimizing extraction processes to obtain polysaccharides with desired properties. Dubois et al. [176] leveraged ^1^H and ^13^C NMR to analyze the composition of gum mucilage extracted from flaxseed. This analysis proved invaluable for comparing compounds extracted through different operating modes and parameters, thereby aiding in the optimization of extraction methods and the understanding of how processing affects the final polysaccharide product [176]. Ultimately, the comprehensive structural and compositional information gleaned from NMR makes it an indispensable technique for advancing the utilization of polysaccharides in diverse fields, from food science to biomaterials.

## 5. Application of Polysaccharides

**Figure 6:**
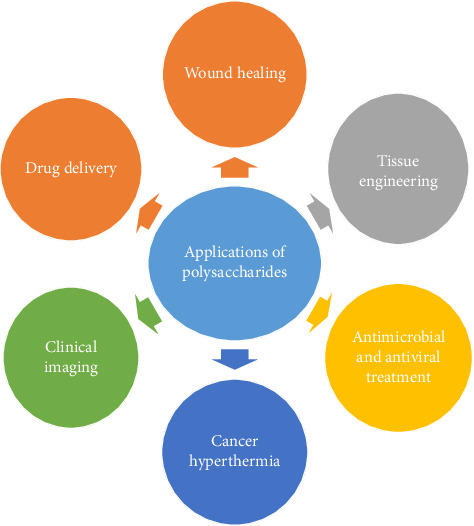
Applications of polysaccharides.

The polysaccharides and their derivatives have more advantages than the synthetic polymers because, when they are compared with the non-natural polymer equivalence, they have vast applications, since they have shown to be less costly, less toxic, biocompatible, more biodegradable. The benefits derived from polysaccharides and their derivatives could be applied to a wide range of fields, that include biomedical research, pharmaceutical development, food science, and cosmetics [177]. In addition to that, with the help of modern technology, the application of polysaccharides nowadays is slowly expanding to more areas such as disease control and health care with the use of traditional herbs, tissue engineering, treatment of the wound, drug delivery, bacterial and viral treatment, and helping in diagnose and prevention of cancers [178]. A comprehensive analysis of key polysaccharides and their specific roles can be found in detail in Table 3, providing a deeper insight into their practical implementations across various fields. Applications of polysaccharides are given in Figure 6.

### 5.1. Tissue Engineering

**Figure 7:**
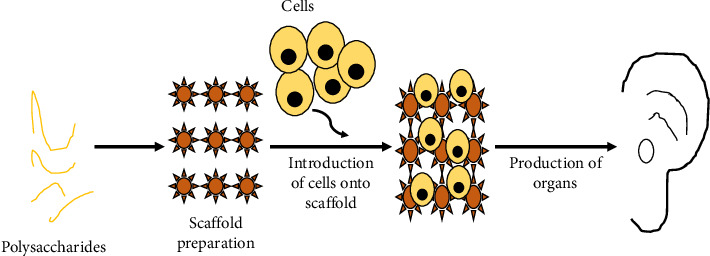
Tissue engineering using polysaccharides.

Polysaccharides and their derivatives are increasingly vital in medical tissue engineering, where biological signaling, cell proliferation, differentiation, and remodeling are crucial for tissue regeneration [179]. These biomaterials, including chitin, cellulose, alginate, chitosan, CS, hyaluronic acid, and starch [180], serve as scaffolds that promote tissue repair. However, they must meet stringent criteria such as biocompatibility, biodegradability with controlled degradation, structural integrity, high porosity with appropriate pore size distribution, and nontoxicity [181, 182]. Chitin and chitosan, for example, possess the properties necessary to mimic tissue scaffolds, particularly in 3D hydrogels or porous sponges, due to their immunogenicity, degradability, and mechanical strength [183]. These 3D scaffolds/structures can be made using the biopolymers and they support the growth of cells, thus making a cell grown organ structure possible (Figure 7). These 3D structures facilitate cell seeding for both in vitro and in vivo studies, supporting cartilage, bone, and tendon regeneration, as well as stem cell encapsulation and differentiation [184]. Notably, chitosan composites combined with hydroxyapatite or carbon nanotubes have shown promise in developing artificial bones and promoting bone regeneration [185, 186].

Beyond chitin and chitosan, other polysaccharides like cellulose, starch, hyaluronic acid, pectins, alginate, agar, dextran, pullulan, gellan, xanthan, and glycosaminoglycans are being explored for their potential in tissue engineering [187–189]. These natural polymers offer distinct biological advantages over synthetic materials, influencing the fabrication and structure of polysaccharide-based materials [190]. Alginate gel matrices have been successfully used for mesenchymal stem cell differentiation into chondrocytes [191] and as a delivery agent for chondrocytes in cartilage construction within nude mouse models [192]. The diverse properties of these polysaccharides enable the creation of tailored scaffolds that support various tissue engineering applications, driving advancements in regenerative medicine.

### 5.2. Wound Healing and Wound Dressing

Polysaccharides like chitosan, chitin, hyaluronan, cellulose, and alginate have gained increasing popularity for their applications in wound healing and dressing. Their widespread use is attributed to their inherent biocompatibility, biomedical activity, natural derivation, cost-effectiveness, and low toxicity levels. Researchers and medical professionals face challenges when developing bioactive materials based on these natural polysaccharides to address various issues related to wound care and management [193–195]. The complexity of wound healing dressing plays a crucial role in the postoperative care of acute and chronic tissue injuries, effectively preventing potential infections caused by bacteria. By serving as a barrier against external environmental agents, wound dressings offer protection to the injury site and mitigate further tissue damage [194]. Taking all of this into account, many researchers have faced challenges in fabricating bioactive materials that are based on numerous natural polysaccharides to tackle wound healing and wound dressing-related problems [196]. A notable example of polysaccharides that can be used in the application of wound healing is hyaluronic acid, which can be used to treat burns, ulcers, traumas, and other deep cutaneous lesions that lead to immense loss of dermis [197].

An illustrative example of a polysaccharide used in wound healing applications is hyaluronic acid, which exhibits exceptional viscoelastic properties and is instrumental in treating burns, ulcers, traumas, and deep cutaneous wounds resulting in dermal loss. Hyaluronic acid's distinct viscoelasticity is akin to its role in synovial fluid and vitreous humor, providing lubrication and shock absorption. The hygroscopic nature of hyaluronic acid creates an osmotic solution, crucial for maintaining tissue hydration, particularly during inflammatory stages postinjury [197, 198].

The development of N, O-carboxymethyl chitosan, nanocurcumin, and oxidized alginate hydrogel showcases promising wound healing capabilities in both in vivo and in vitro settings [199]. Films made using biopolymers have the potential to be biodegradable, biocompatible, non-toxic, and barriers of oxygen and carbon dioxide. They also seem to show good antimicrobial activity. For instance, silver nanoparticles embedded in polyvinyl pyrrolidone and alginate hydrogels are known to prevent fluid accumulation in exuding wounds, a process achievable through gamma radiation [200]. The inclusion of nanosilver with polymers like polyvinyl pyrrolidone and alginate hydrogels confers potent antimicrobial effects, crucial for safeguarding against bacterial infections during wound dressing procedures, making it a viable option for wound healing applications [201, 202].

### 5.3. Drug Delivery and Controlled Release

In pharmaceutical industries, natural polysaccharide polymers are extensively utilized as drug delivery systems, serving as natural excipients whose primary function is to facilitate the delivery of drugs to their intended targets. This advantageous characteristic is especially exploited in the use of plant-derived [203] Polysaccharides due to their inherent properties such as low toxicity, biocompatibility, biodegradability, affordability, and widespread acceptance among the general public.

The significance of drug delivery and controlled release mechanisms in the medical field cannot be overlooked, as they are important to understand the pharmacokinetics of various drugs. Processes like absorption, distribution, metabolism, and excretion govern the movement of drugs within the body. However, numerous challenges impact the effective delivery of drugs to specific disease sites, including issues like poor solubility, enzymatic degradation, rapid renal clearance, unwanted side effects, inadequate cellular uptake, and structural breakdown of drugs in the process of reaching the infected or disease site, and more [204].

**Figure 8:**
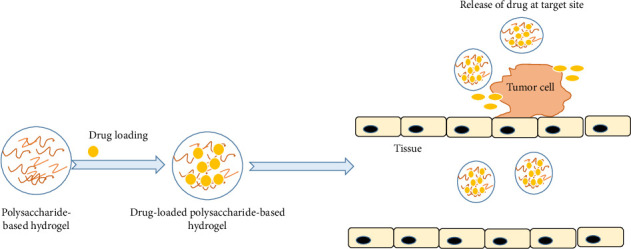
The schematic representation of polysaccharide-based DDS in the treatment of cancer.

Historically, chemotherapy has been a primary treatment approach for cancer patients, despite limited success rates due to the restricted accessibility of drugs to tumor sites. Addressing this limitation through the development of smart polysaccharide-based nanodrug delivery systems offers promising solutions for treating both noninfectious diseases and cancer [120, 205]. These innovative systems enable the customization of drug size and structure to facilitate crossing small capillary walls and evade clearance by the mononuclear phagocyte system, thereby prolonging drug circulation time in the bloodstream. Leveraging the enhanced permeability and retention effects of large nanoparticles and macromolecules improves the localization of nanomedicine at tumor sites, resulting in more effective treatment compared to smaller nanoparticles and low-molecular-weight compounds (Figure 8) [206].

Moreover, researchers are increasingly exploring the immune-stimulating properties of polysaccharides derived from various plants and herbs as an alternative treatment for infectious diseases and cancer. Active compounds found in traditional Chinese medicine polysaccharides have shown the ability to enhance interleukin activity, raise antibody levels, modulate T cells and macrophages, and regulate the immune response in organisms, highlighting their potential therapeutic benefits [20].

In the current development, researchers have made significant strides in achieving controlled release of anticancer drugs through the utilization of starch-based materials that have been specifically modified by hydrogel, polymeric capsules, graft, and polymeric polyethylene glycol. These modifications are designed to enhance the efficacy of antimetastasis and anticancer treatments while mitigating toxicity in patients with prostate cancer [207]. Among the starch-based materials used are pH-responsive hydroxyethyl starch, which has been conjugated with doxorubicin or luteinizing hormone-releasing hormone for targeted delivery. Moreover, potato starch has also shown promise in therapeutic and in vivo diagnostic applications. The development of potato starch-based microparticulate systems through oxidation and polyamine integration has opened new possibilities for treatment strategies [208].

Doxorubicin remains a cornerstone chemotherapy agent for various types of cancer, including ovarian, lung, bladder, and breast cancer [209]. Starch grafted with polyethylene glycol copolymers has emerged as a method for enhancing the delivery of doxorubicin, particularly by facilitating the intracellular response of glutathione to the drug [210]. These advancements in starch-based materials exemplify the ongoing innovation and versatility in drug delivery systems for combating cancer effectively. One achievable hemostatic substance is chitosan. Chitosan has a positive charge that can aggregate the red blood cells, which is a negatively charged, and that will improve the platelet adhesion that is linked to the hemostatic action and aids in blood coagulation. An experiment which was conducted in vivo liver bleeding shows the chitosan nanofibers. This chitosan nanofiber has high hemostatic performance and a high degree of biodegradation at the last stage of surgery. Thus, the results obtained proved the viability and huge application potential in the treatment of bleeding wound [211].

**Figure 9:**
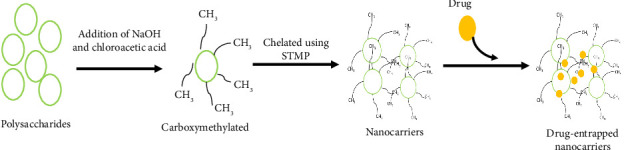
Production of polysaccharide-based nanocarriers.

Purified polysaccharide-derived animals or plants can be used for the synthesis of nanocarriers [212–214]. Plant gum-derived polysaccharides are required to be carboxymethylated before forming a nanocarrier, as this carboxy methylation favors the chelation using sodium trimetaphosphate (STMP) [49] (Figure 9). Similarly, enhanced delivery of doxorubicin in MCF7 breast cancer cells was observed due to the use of polyelectrolyte magnetic nanocarrier that was developed using carboxymethyl cellulose (CMC) polymer which resulted in the reduced side effect of the drug [215]. In another study, the formation of core–shell polyelectrolyte complexes was achieved by incorporating zinc oxide nanoparticles with CMC beads and a layer of chitosan through a self-assembly technique. They were found to be efficient carriers of colon-specific drugs and also exhibited pH sensitivity when loaded with anticancer drugs, 5-fluorouracil (5-FU) [216]. Mesalamine (5-aminosalicylic acid) is an anti-inflammatory drug that is utilized in treating bowel disease or Crohn's disease, but its major disadvantage is its solubility of the aqueous system which causes effortless permeability of the drug into the gastrointestinal tract, leading to a decreased amount of drug reaching the target area. This was overcome by CMC–rosin gum hybrid nanoparticles that were synthesized via nanoprecipitation method, and it was observed that 72% of the loaded drug was released in a controlled manner in contrast to the native CMC or rosin gum [217]. The use of polysaccharides in drug delivery is advantageous as they cause improved bioavailability, stability, and reduction of side effects [218]. The advantage of polysaccharides also makes an efficient drug delivery vehicle for cancer treatment. Due to their optimal size and surface properties, the polysaccharide-mediated nanocarriers can be engineered for targeted action on tumor cells and increased circulation time in the bloodstream. Therefore, this can increase the targeting efficacy of chemotherapeutics resulting in lower side effects [219]. Polysaccharide-based nanoparticles also facilitate oral administration of noninvasive drugs, and they protect bioactive compounds against enzymatic degradation due to the protective polymer matrix [220]. The distinctiveness of polysaccharide nanocarriers is that they are hydrophilic, and the bonds present in them form noncovalent bonds with biological tissues such as epithelial and mucoadhesive [221]. Certain cases like premature drug release, lack of cytospecificity, and shorter time blood circulation have been a biggest challenge. To overcome these complexes, different polysaccharide-dependent nanoformulation has been developed not only handle above-mentioned problems but also minimize the premature degradation of drugs. So a polysaccharide-based formulation drug has been developed in order enhance this issue, as this drugs can release different stimuli based on the composition of the developed system [222].

Various forms of polysaccharides act as efficient drug delivery systems. For instance, starch is one of the most readily available polysaccharides that is extensively being used for the synthesis of nanocarriers. Acha (*Digitaria exilis*) starches have been utilized to synthesize nanocarriers for poorly soluble drugs (naproxen). Carboxymethylation was done by hydrolysis of starch with H2SO4 and treating it with monochloroacetic acid and sodium hydroxide. Seventy-five percent of the loading efficiency of the drug and sustained release of naproxen were observed [223]. Likewise, a nonionic polysaccharide that is derived from seeds of *Cyamopsis tetragonolobus*, known as guar gum, has been utilized for the synthesis of nanocarriers due to its disintegrant and binding properties. Chitosan nanoparticles synthesized using ionotropic gelation–spray drying technique were coated with guar gum and were studied for therapeutic potential against tuberculosis by loading the nanocarriers with the antitubercular drug. It was found that the material had the highest cell uptake potential along with the pattern of sustained release of the drug observed through a biphasic pattern of in vitro drug release behavior. Furthermore, histopathology studies revealed no lung tissue abnormality, thus proving to be a promising drug carrier with minimal side effects [224]. Purified polysaccharide derived from *Araucaria heterophylla* was subjected for carboxymethylation where it was chelated using STMP to form nanocarriers and further loaded with curcumin to observe the in vitro cytotoxic effect against the MCF7 human breast cancer cell lines. MTT assay revealed the presence of having a significant cytotoxic effect against the cancer lines, which may be taken as evidence of the release of drug within the cell. It was also proposed that the enhanced activity was due to the anticancer nature of the polysaccharide [225].

### 5.4. Food Industry

The contribution of polysaccharides in the food industry has been gaining a lot of importance as they are carbon sources abundantly found in the biosphere. They are utilized in their natural forms, but they can also be chemically modified for adaptability such as improved solubility and water binding capacity [226]. They contribute to the food industry in different ways by acting as a food preservative, food packaging, food additives/nutrition, and so on. For instance, carbohydrate polymers are widely used in food processing and packaging due to their ability to produce the physical stability required for the packaging and distribution of food through emulsions or suspensions [227]. A marine polysaccharide derived from red seaweeds is known as agar and is extensively used in the food industry due to its advantage of gelation at low temperatures like 30°C–40°C [228]. Similarly, various plant-derived gums such as tamarind gum and xanthum gum also play an important role in the food industry as they modify the behavior of water in the food systems to provide control of crystal size in saturated sugar solutions [229]. Given that it strengthens and maintains the food's structure, as additions, they are widely used in culinary preparations such as jams, jellies, ice cream, and other milk products. The most widely used and commercially significant SP in the food processing industries are sulfated galactans, such as carrageenan and agar [230].

Certain polysaccharides that are derived from plants or microbial sources are also used as additives to provide nutrition in the food industry. For instance, a microbial polysaccharide obtained from *Sphingomonas paucimobilis* (formerly known as *Pseudomonas paucimobilis*) is utilized as a stabilizer, gelling agent, and thickening agent in many food sources due to its unique structure and texture in many food substances. It can also be used as an alternative to gelatin. It is mainly utilized in modified starch to improve its ability as a stabilizer and water binding agent, and additionally, preventing the “blunting effect” of starch observed on the flavor of the food [231]. A linear polysaccharide known as dextran is obtained from *Leuconostoc mesenteroides* and *Acetobacter Sp*. is reported to be the first commercialized microbial polysaccharide that is approved to be used in the food industry. It is mainly used as a gelling agent in gels and in sweetening agents to retain moisture, viscosity, and to inhibit sugar crystallization [232]. Chitosan, a deacetylated derivative of chitin, is the second most abundant polysaccharide found in nature. Certain studies suggest that chitosan can be used as an alternative to packaging materials for quality preservation of food materials and is proven to be advantageous due to its antibacterial property bivalent mineral chelating ability [233, 234]. A mixture of chitosan–tapioca starch was observed for its antimicrobial activity of edible coating solutions with and without the addition of potassium sorbate. An inhibitory effect of chitosan was observed against *Lactobacillus spp* through agar well diffusion assay, with the presence of potassium sorbate and/or tapioca starch. A salmon slice coating assay revealed reduced mesophilic and psychrophilic cell count was observed due to chitosan coating, along with reduced water vapor permeability and solubility of starch films [235]. Thus, various polysaccharides have been utilized in the food industry in different ways due to their biodegradable and nontoxic property.

Table 4 comprises a brief evaluation of various polysaccharides and their applications.

## 6. Conclusion

Naturally derived polysaccharides, including chitosan, plant gums, cellulose, and starch, represent an abundant and invaluable class of biopolymers with diverse applications across biological and medical fields. This review provided a comprehensive overview of these materials, detailing their extraction and purification procedures. The wide array of characterization techniques employed, such as UV–Vis spectroscopy, FTIR, SEM, TEM, and XRD, and how these inform their potential applications, was well discussed. These polysaccharides are particularly notable for their exceptional intrinsic properties, which are highly exploitable for advanced medical applications. Their inherent biocompatibility, biodegradability, antimicrobial, antioxidant, and anti-inflammatory properties make them ideal candidates for innovative therapeutic strategies, including their significant utility in nano/micro-based targeted drug delivery systems. Over recent decades, bioderived polysaccharides have profoundly impacted the development and progression of fields like nanotechnology, tissue engineering, and nanomedicine. The ongoing investigation into novel methodologies for polysaccharide utilization holds immense future potential, promising new avenues for treating diseases and significantly enhancing overall public health.

## Figures and Tables

**Table 1:** Merits and demerits of various extraction methods.

Extraction method		Advantages	Disadvantages	References
Chemical method	Alkaline-based water extraction	High extraction rate, less time-consuming, acidic polysaccharides can be extracted, easy to operate	Poor selectivity, low purity, polysaccharide degradation	[91]
	Acid-based water extraction	High extraction rate, low impurity content	The polysaccharide structure may be destroyed	[92]

Physical method	Ultrasonic-assisted extraction	Rapid and effective method, easy operation, high extraction efficiency, no damage to the active ingredients, environment-friendly method	Amount of impurities is relatively high, ultrasonic power applied must be high for high extraction rate	[92]
	Microwave-assisted extraction	Improves the yield of polysaccharide, reduces solvent usage	High capital cost	[92]
	Infrared-assisted extraction	Short extraction time, offers high permeability, low-temperature operation, free irradiation, and low cost.	Still in research stage	[40, 72]
	Dynamic high-pressure microjet technology	High rate of extraction, less extraction time	May lead to polysaccharide chain breakage and can change its original characteristics	[40, 84]
	Subcritical water extraction	Helps to enhance rate of extraction when combined with other extraction methods	Still in basic research stage	[40, 65]
	Supercritical CO_2_ extraction	Ecofriendly method, can be used for thermolabile components	Requires high pressure, high-cost investment	[92]

Biological method	Enzymatic hydrolysis	Reaction conditions are mild	Seldom used alone; usually combined with other extraction methods for efficient extraction	[93]

**Table 2:** Merits and demerits of various purification methods.

Purification method		Advantages	Disadvantages	References
Deproteination of polysaccharides	Sevag method	Most commonly employed method, easy operation	Is effective in removing free proteins rather than protein–polysaccharide complex from crude polysaccharides	[96]
	TCA precipitation method	Has the ability to remove total proteins from crude polysaccharide	The rate of polysaccharides loss is less when compared to other deproteination processes	[96]

Chromatography column separation	Anionic exchange column chromatography	Fit for purifying various acidic/neutral polysaccharides and mucopolysaccharides, widely used technique, possess a large separation capacity	High cost, sometimes the height of column bed may change when pH of buffer changes, the flow rate of eluent is easily affected by the changes of volumes, which is sensitive with the changes of eluent pH or the ion strength of solution	[40]
	Gel column chromatography	Quick, convenient, and effective separation process	The ionic strength of eluent should not be less than 0.2 mol/L, unsuitable for the separation of mucopolysaccharides	[40]
	Affinity column chromatography	High efficiency, easy-to-operate, can separate polysaccharides with less content, one-time enrichment of polysaccharides is very high	Very difficult to find a proper and suitable ligand for a given polysaccharide	[40]

Centrifugation		Effective separation, removal of insoluble debris, scalability, ease of handling	Limited separation of similar-sized molecules, potential for degradation, difficulty in separating soluble impurities	[101]

Ultrafiltration		Can simultaneously concentrate and purify, high-throughput, mild operating conditions so degradation possibility is low	Low speed, time-consuming, possibility of degradation, membranes may adsorb the polysaccharides	[101]

Dialysis		Gentle separation, versatility, effective removal of small molecules	Limited separation of similar-sized molecules, may result in dilution, time-consuming	[101]

**Table 3:** Chemical bonds observed in polysaccharides.

Source	Type of polysaccharide	Peak position (cm^−1^)	Functional groups	References
Cedar wood	Cellulose	3000–2800	C–H aliphatic stretching vibration of the primary, secondary, and tertiary alkyls groups	[129]
		2919	Asymmetric stretching vibration *v*vasCH2 and *v*vCH3	

*Albizia zygia*	Plant gum	703.08	C–H bending, phenyl ring substitution band	[127]
		933.58	C–H bending, alkene	
		1084.03	C–O stretching, carboxylic acid, ether, ester, alcohol	
		1321.28	C–N stretching, amine	
		1423.51	C–H scissoring and bending, alkanes	

Cladophora sp.,	Cellulose	3332	-OH groups stretching vibration	[130]
		2914	C–H stretching vibration	
		1640	H2O absorbed	
		1423	CH2 bending vibration	
		909	C–H rock vibration	

Tamarind gum	Plant gum	3401	O–H stretching	[131]
		2925	CH and CH2 stretching	
		1647	Carboxylate ion stretching	
*Acacia senegal*	Plant gum	3438	O–H stretching	
		2925	CH and CH2 stretching	
		1630	Carboxylate ion stretching	

American cockroach	Chitosan	3400	O–H stretching	[132]
		2923.88	C–H stretching	
		1650.95	C=O stretching	
		894.91	β-1-4 glycosidic linkage	

Ginger essential oil	Starch, chitosan	3000–3600	O–H and N–H oscillations	[122]
		2850–2960, 1377	C–H stretching	
		3550–3096	O–H and N–H stretching	

Fungi from pineapple peel	Chitosan	2921.73 and 2850.33	C–H stretching	[123]
		1638.11	N–H bending	
		1022.46	C–O stretching	

Chitosan–hesperidin nanoparticles	Chitosan	1600	NH–H bending	[121]
		1670	Amide II carbonyl	
		2923.09	O–H stretching	
		1644.35	Carbonyl groups	
		1517.22	Aromatic rings	
		1030	Alcohol bands	
		1070	C–N bands	
		2918 and 2849	C–H bands	

**Table 4:** Application of various bio-derived polysaccharides.

Name of the polysaccharide	Type of polysaccharide	Application	Reference
Starch	Homopolysaccharide	Widely used in spray drying, extrusion, molecular inclusion, coacervation with proteins, hydrocolloid-forming, release via swelling, diffusion, and erosion; acts as thickener, stabilizer of frozen food, encapsulation of flavors in dairy products, agriculture mulch films, diluent, and carrier in toilet powders	[236–240]
Cellulose	Homopolysaccharide	Thickener, emulsion stabilizer, film forming, and surfactant properties; noncaloric bulking agent and binder in prepared foods; prevention of ice crystal growth in frozen foods, used in compression tablets, syrups, granules, semisolid preparations, transdermal patches; osmotic and enteric-coated drug delivery systems; bioadhesive formulations (buccal, nasal, transdermal, vaginal); drugs and liposomal formulations, water remediation, and facial moisturizer	[241–245]
Glycogen	Homopolysaccharide	Has film-forming capacities, used as bioactive or antioxidant films for food applications, used as collagen crosslinker to obtain matrices with defined crosslinking degrees	[246]
Hyaluronic acid	Heteropolysaccharide	Vital application in the field of oncology, orthopedics, ophthalmology, osteopathic perspective, fascial techniques aiming to release tensions, reduction of pain, and restoration of function based on the viscoelastic properties in the connective tissue	[247–249]
Heparin	Heteropolysaccharide	Antibacterial, antimicrobial properties, and AgNPs-loaded heparin polyelectrolyte for the microbial infection in dental implants	[250]
Chondroitin sulfate	Heteropolysaccharide	Delivery system, used in tissue engineering	[251]
Chitosan	Homopolysaccharide	Antifungal, antibacterial, reduces LDL (low-density lipoprotein), tissue regenerative, pulmonary delivery ionotropic gelation, coacervation with anions, modified emulsification, antimicrobial and preservative agent, edible film, Accelerator of calcium and iron absorption, dietary fiber, protective effect on bacterial infection, immuno-enhancing and antitumor agent, antiulcer, and antiacid action. Acts as a carrier for drug delivery antioxidant, accelerator of wound healing, support for immobilized enzymes, porous beads for bioreactors and membrane material, wastewater treatment. Seed-coating preparation, activator for plant cells, water-retaining and moisturizing agent for hand and body creams, shampoos, and toothpastes	[21, 200, 252–261]
Dextran	Homopolysaccharide	Used as a viscosifier. A film of dextran is used in frozen foods, especially in ice cream, colon-targeted delivery, formation of porous particle pulmonary delivery	[262, 263]
Fructan	Homopolysaccharide	*Asparagus* production, controls regrowth and postharvesting sprouting, acts as prebiotic	[264, 265]

## Data Availability

No data were used in this review, as it is based solely on the published literature.
